# Role of Transcriptional Read-Through in PRE Activity in Drosophila melanogaster

**Published:** 2016

**Authors:** P. V. Elizar’ev, D. V. Lomaev, D. A. Chetverina, P. G. Georgiev, M. M. Erokhin

**Affiliations:** Institute of Gene Biology, Russian Academy of Sciences, Vavilov str. 34/5, 119334, Moscow, Russia

**Keywords:** Polycomb, Trithorax, PRE, Drosophila

## Abstract

Maintenance of the individual patterns of gene expression in different cell types is required for the differentiation and development of multicellular organisms. Expression of many genes is controlled by Polycomb (PcG) and Trithorax (TrxG) group proteins that act through association with chromatin. PcG/TrxG are assembled on the DNA sequences termed PREs (Polycomb Response Elements), the activity of which can be modulated and switched from repression to activation. In this study, we analyzed the influence of transcriptional read-through on PRE activity switch mediated by the yeast activator GAL4. We show that a transcription terminator inserted between the promoter and PRE doesn’t prevent switching of PRE activity from repression to activation. We demonstrate that, independently of PRE orientation, high levels of transcription fail to dislodge PcG/TrxG proteins from PRE in the absence of a terminator. Thus, transcription is not the main factor required for PRE activity switch.

## INTRODUCTION

During the early stages of development of multicellular organisms, an individual pattern of gene expression is established in different cell types and then maintained over many cell divisions. Polycomb (PcG) and Trithorax (TrxG) group proteins are responsible for a stable inheritance of the proper pattern. PcG proteins cause repression, while TrxG proteins provide activation of transcription [1-4]. In *Drosophila*, these factors bind to DNA elements called PREs (Polycomb Response Elements). PRE elements contain sites for various DNA-binding factors, the recruitment of which results in association of PcG/TrxG complexes with PRE [5, 6]. Polycomb group proteins are assembled into three main complexes: PRC1, PRC2, and PhoRC [2, 3]. The core subunits of the PRC1 complex are represented by the PC, PH, dRing, and Psc factors [7-9]. The PRC2 complex contains the E(z), Esc, Su(z)12, and Caf1 core components [10-13]. The PhoRC complex includes the dSfmbt and DNA-binding factor Pho [14]. PRC2 complex trimethylates lysine 27 of histone H3 (H3K27me3) through the SET domain of the E(z) catalytic subunit [10-13]. H3K27me3 modification specifically marks the chromatin regions repressed by PcG [15, 16]. TrxG proteins represent a heterogeneous group which in particular includes the Trx, Trr, dCBP, Ash1, and UTX factors and DNA-binding factor GAF, also known as Trl (Trithorax-like) [17].

The activity of PREs can be modulated. For example, the repressor activity of PREs in transgenic systems can be turned off either by enhancers or the yeast exogenous activator GAL4 [18-24]. It has been previously suggested that inactivation of repression is provided by the induction of transcription through PRE by the GAL4 activator, which, in turn, leads to the removal of PRE-associated repressor factors from DNA due to the passage of RNA polymerase II and transcription factors [24].

However, we have recently demonstrated that even a high level of transcription through the 660 bp* bxd*PRE does not lead to complete elimination of proteins from *bxd*PRE in *Drosophila *transgenic constructs [21]. Transcription was initiated from the UAS-promoter: the minimal promoter of the *hsp70 *gene under the control of five binding sites for the GAL4 protein. We showed that inactivation of PRE-mediated repression was independent of whether GAL4-induced transcription was directed towards or in opposite direction from* bxd*PRE.

In the present study, we show that prevention of transcription through *bxd*PRE by a SV40 terminator does not abrogate inactivation of PRE-mediated repression. The importance of *bxd*PRE orientation in transcriptional read-through has been also tested. It has been established that, in case of reverse orientation of *bxd*PRE, transcriptional read-through also does not lead to the elimination of PcG/TrxG factors.

## EXPERIMENTAL

**Plasmid constructs design**

All constructs were made on the basis of the CaSpeR vector containing *white *gene with partial deletion of the first intron (encodes complete product of *white* gene) [25]. The enhancer of *white *gene (Ee) located in the genome at position –1180…–849 bp relative to the transcription start site of *white *gene [26] was excised from the Ee-pBluescript SK+ plasmid [27] and inserted in forward orientation into a CaSpeR4 vector cleaved by NotI [En-*white*].

A fragment SmaI-SalI of 4324 bp in length from the plasmid vector CaSpeR-hs43-lacZ carrying the *lacZ* gene with the *adh *leader sequence and SV40 transcription terminator at 3'-terminus (GenBank: X81643.1) was inserted into a pBluescript SK+ vector cleaved by SmaI and SalI [LacZ-SV40-pSK].

The promoter of the *hsp26 *gene, 472 bp, was amplified by PCR (primers 5'-ctagaaacttcggctctctca-3' and 5'-gttgaatgaacttgtttgacttgt-3') and inserted into a pBluescript SK+ vector cleaved by EcoRV [hsp26- pSK]. A HindIII–PstI fragment of the hsp26-pSK vector was inserted into the LacZ-SV40-pSK vector at the SmaI site [hsp26-LacZ-SV40-pSK]. A fragment NotI-SalI of the hsp26-LacZ-SV40-pSK vector was incorporated into the En-*white *vector at the BamHI site [hsp26-LacZ-SV40-En-*white*].

A fragment HindIII–EcoRI containing a minimal promoter of the *hsp70 *gene and five GAL4 sites at 5' terminus was excised from the pUAST vector [28] and inserted into a pBluescript SK+-sce2 vector at the EcoRV site [sce(UAS)]. The coding region of a *eGFP* gene of 717 bp was amplified by PCR (primers 5'-atggtgagcaagggcgaggagct- 3' and 5'-cttgtacagctcgtccatgccga- 3') and cloned into the vector pBluescript SK+ at the EcoRV site [eGFP-pSK].

A HindIII–EcoRI fragment of the eGFP-pSK vector was inserted in forward orientation into the sce(UAS) vector at the HincII site [(UAS)sce-eGFP].

A XbaI–BamHI fragment of 702 bp in length of the pUAST vector containing a transcription terminator was inserted into a pBluescript SK+-lox2 vector cleaved by EcoRV [lox(SV40)]. A XbaI–XbaI fragment of the lox(SV40) vector was incorporated into the (UAS)sce-eGFP vector at the XhoI site [(UAS) sce-eGFP-lox(SV40)].

A HincII–HincII fragment, 1828 bp, of the LacZSV40- pSK vector was incorporated into a pBluescript SK+ vector cleaved by EcoRV [*linker*1828bp-pSK]. A fragment XbaI–BamHI of 222 bp of the pGL3basic vector containing the SV40 transcription terminator was inserted into the *linker*1828bp-pSK vector at the SmaI site [*linker1828*-SV40s-pSK].

A fragment NotI–BamHI of the (UAS) sce-eGFP-lox(SV40) vector was inserted into the vector* linker1828*-SV40s-pSK at the EcoRV site [(UAS) sce-eGFP-lox(SV40)-*linker1828*-SV40s-pSK].

A fragment HincII–XbaI containing *bxd*PRE of 656 bp (3R:16764122..16764777) was excised from the frt(PRE) vector [29] and incorporated into the vector (UAS)sce-eGFP-lox(SV40)-*linker1828*-SV40s-pSK at the AorI site in forward [(UAS)sce-eGFP-lox-(SV40)-linker785frt(PREdir)linker1043-SV40s-pSK] or reverse [(UAS)sce-eGFP-lox(SV40)-linker785frt(PRErev)linker1043-SV40s-pSK] orientation.

*UDTPD construct. *A XbaI–XbaI fragment of the (UAS)sce-eGFP-lox(SV40)-linker785frt(PREdir)linker1043- SV40s-pSK vector was incorporated into the vector hsp26-LacZ-SV40-En-white at the BamHI site.

*UDTPR construct*. A XbaI–XbaI fragment of the (UAS) sce-eGFP-lox(SV40)-linker785frt(PRErev)linker1043-SV40s-pSK vector was inserted into the hsp26-LacZSV40-En-white vector at the BamHI site.

All details of the constructs design are available upon request.

**Transformation of *Drosophila melanogaster* embryos and phenotypic analysis of *yellow* and *white *expression in transgenic lines**

DNA constructs and a P element with defective inverted repeats *P25.7wc*, which served as a source of transposase [30], were injected into a *y^1^w^1118^*line at the stage of preblastodermal embryo according to [31, 32]. The survived flies were crossed with the *y^1^w^1118^*line. Transgenic flies were selected based on phenotypic manifestation of *white *expression. The number of copies was determined by Southern blot hybridization with a* white *gene fragment. Lines containing a single copy of the construct per genome were selected.

For *in vivo *deletion of the DNA fragment, flies carrying the construct were crossed with transgenic flies expressing Flp (*w^1118^; S2CyO, hsFLP, ISA/Sco; +*) or Cre (*y1w1; Cyo, P[w+,cre]/Sco; +*) recombinase [33, 34]. Accuracy of fragment removal was confirmed by PCR.

Line *yw^1118^*; *P[w^-^P, tubGAL4]117/TM3,Sb, *a derivative of the Bloomington Stock Center #5138 line with deletion of the *mini-white *marker gene [35], was used for expression of *GAL4 *under the control of a tubulin promoter.

The expression of *white *gene was determined by visual evaluation of eye pigmentation using the standard scale: red color is the pigmentation of eyes in wildtype flies (*white *expression in case of complete stimulation by a tissue-specific enhancer), white the color of the eyes is observed in the absence of pigmentation (complete inactivation of *white *gene). Various degrees of mosaic phenotype are observed in case of repression.

In order to analyze the phenotype of transgenic flies, 3- to 5-day-old males developed at 25°C were used. The details of all crosses conducted for the genetic analysis and excision of functional elements can be provided upon request.

**Chromatin immunoprecipitation (X-ChIP)**

A total of 150–200 mg of adult flies was collected for each experiment. Chromatin immunoprecipitation was performed according to the technique described previously [21].

**Antibodies**

Antibodies to the PH protein [to fragment 86–520 aa, ph-p-PA]; dSfmbt [to fragment 1–348 aa, Sfmbt-PB] [27]; PC [to fragment 191–354 aa, Pc-PA]; TRX-N [to fragment 8–351 aa, trx-PA]; and GAF [1–519 aa, Trl-PB] [21] were obtained in rabbits. Antibodies to H3K27me3: Abcam (ab6002, ChIP Grade).

**Real-time PCR with Hot-Start Taq DNA polymerase**

Real-time PCR was conducted using C1000tm ThermalCycler (Bio-Rad) in a 25 •µl volume according to the following protocol (per one reaction): 2.5 •µl of 10× buffer (0.5 M Tris-HCl, pH 8.8, 0.5 M KCl, 15 mM MgCl_2_, 1% Tween 20), 2 •µl of 25 mM MgCl_2_, 0.5 •µl of 10 mM dNTPs, 1.5 •µl of each primer (at a concentration of 5 pmol/ •µl), 0.25 •µl of SYBR Green100× (Sigma), 0.3 •µl of Hot-Start Taq DNA polymerase (SibEnzyme), 11.45 •µl of mQ, 5 •µl of sample. Data were assessed using the Bio-Rad CFX Manager software and Microsoft Excel. Decimal dilutions of *Drosophila *genomic DNA at a concentration of 0.1 to 100 ng were used as reference standards. The primers used for real-time PCR analysis of the material obtained using chromatin immunoprecipitation are presented in *Table*.

**Table:** Primers for real-time PCR analysis of the material obtained by chromatin immunoprecipitation

1-ChIP forward	5’-gagaactctgaatagggaattgg-3’
1-ChIP reverse	5’-agctcctcgcccttgctcaccat-3’
2-ChIP forward	5’-ccgaccactaccagcagaac-3’
2-ChIP reverse	5’-gtccatgccgagagtgatcc-3’
3-ChIP forward	5’-tcctcgacggtatcgataagcttg-3’
3-ChIP reverse	5’-ccataatggctgcgccgtaaag-3’
4-ChIP forward	5’-ggtgaaattatcgatgagcgtgg-3’
4-ChIP reverse	5’-cagttcaaccaccgcacgataga-3’
5-ChIP forward	5’-aaaactttctacgcctcagttc-3’
5-ChIP reverse	5’-gcttattagccctgcaattga-3’
6-ChIP forward	5’-gcactggatatcattgaacttatctg-3’
6-ChIP reverse	5’-tggacagagaaggaggcaaaca-3’
Ras64B forward	5’-gagggattcctgctcgtcttcg-3’
Ras64B reverse	5’-gtcgcacttgttacccaccatc-3’
bxdPRE adjacent forward (site adjacent to bxdPRE in genome)	5’-aagagcaaggcgaaagagagc-3’
bxdPRE adjacent reverse (site adjacent to bxdPRE in genome)	5’-cgttttaagtgcgactgagatgg-3’

## RESULTS

**Model system for studying the impact of transcription on the recruitment of Polycomb and Trithorax group proteins to PRE**

The influence of transcriptional read-through on PRE activity was studied using transgenic constructs integrated into the *D. melanogaster *genome by microinjection of embryos with plasmid DNA due to the 5’ and 3’ termini of the P element flanking the transgene. The 660 bp *bxd*PRE element from the regulatory region of the *Ubx *gene was used [36, 37]. This PRE element is well studied and has binding sites for various PcG and TrxG proteins [15, 21, 36, 37].

**Fig. 1:**
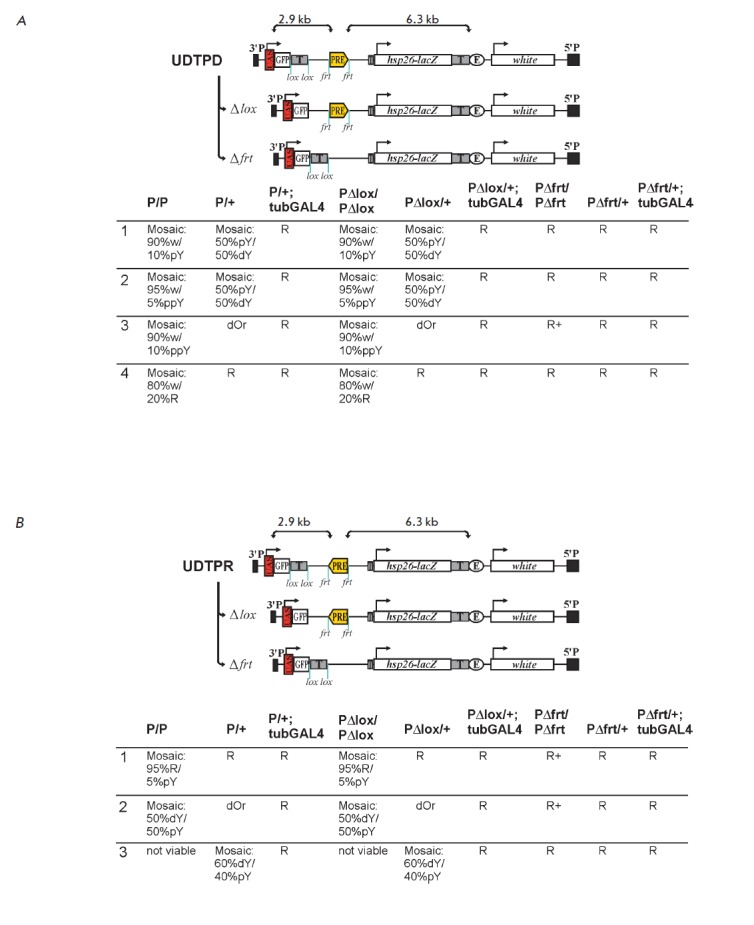
Schematic representation of transgenic constructs and phenotype analysis of flies. A – UDTPD transgene. Minimal promoter of hsp70 gene under the control of binding sites for activator protein GAL4 (UAS) triggers transcription through eGFP and bxdPRE “T” – transcription terminator; hsp26-lacZ and white – reporter genes; “E” – white gene enhancer. Phenotypes of the obtained lines are shown below. P/P – homozygous line; P/+ – heterozygous line; P/+ tubGAL4 – heterozygous line expressing GAL4; PΔlox/PΔlox – homozygous line carrying a deletion of the transcription terminator; PΔlox/+ – heterozygous line with deletion of the transcription terminator; PΔlox/+ tubGAL4 – heterozygous line expressing the GAL4 protein with deletion of the terminator; PΔfrt/PΔfrt – homozygous line with deletion of bxdPRE; PΔfrt/+ – heterozygous line with deletion of PRE; PΔfrt/+ tubGAL4 – heterozygous line expressing GAL4 with deletion of bxdPRE. Scale of eye pigmentation depending on the level of white expression: R+ – dark red (wildtype); R – red; BrR – brownish-red; Br – brown; dOr – dark orange; Or – orange; dY – dark yellow; pY – pale yellow; ppY – very light yellow; w – white. Mosaic pigmentation of eyes is indicated as mosaic. B – UDTPR transgene

Two constructs were created containing *bxd*PRE inserted between the UAS promoter and the reporter genes: *lacZ *under the control of the *hsp26 *gene promoter and *white *gene. The marker *white *gene is responsible for eye pigmentation. Increased level of *white* gene expression in the eyes of flies was obtained by insertion of a tissue-specific enhancer directly upstream of the *white *promoter. The UAS promoter used for induction of transcription through *bxd*PRE is the minimal promoter of the *hsp70 *gene with five upstream binding sites for yeast GAL4 activator. A high level of transcription is achieved upon induction of the UAS promoter (by crossing transgenic lines with a line carrying the *GAL4 *gene under the control of the tubulin promoter). In both constructs, the UAS promoter is directed towards *bxd*PRE. However, the first construct (UDTPD) carries *bxd*PRE in forward orientation, while in the second construct (UDTPR) *bxd*PRE is located in reverse orientation relative to the UAS promoter (*Fig. 1*). In order to suppress internal transcripts of the transgene, two SV40 terminators were used: upstream of the *hsp26-lacZ *gene and upstream of the *white *gene enhancer. An additional transcription terminator, SV40, was inserted at the 5’ side of *bxd*PRE in order to block transcription from the UAS promoter.

Key elements, *bxd*PRE and the SV40 terminator at the 5’ side of *bxd*PRE in both constructs, were flanked by the LOX or FRT site for site-specific recombinases Cre or Flp, respectively. This approach allows one to excise *in vivo *the selected DNA fragments and to compare the expression of the marker gene and functional changes in the system in the presence or absence of key elements at the same genome position (sites of transgene integration).

As a result of construct transformation, four independent transgenic lines for UDTPD (*Fig. 1A*) and three lines for UDTPR (*Fig. 1B*) were obtained with* bxd*PRE in repressed state. Repression of the *white* gene was enhanced in homozygous flies. This effect is characteristic of PRE elements and called PSS (Pairing Sensitive Silencing) [38]. The phenotypes of the UDTPD and UDTPR transgenes were similar; i.e., the effects were independent of *bxd*PRE orientation. Deletion of the transcription terminator located between the UAS promoter and PRE did not result in any phenotypic changes. However, the induction of the UAS promoter by GAL4 led to derepression of the *white* gene both in the case of terminator deletion and in intact lines. Thus, GAL4 inactivates *bxd*PRE in the studied system regardless of orientation and presence of a terminator between the UAS promoter and *bxd*PRE.

**Transcription through *bxd*PRE does not lead to elimination of Polycomb and Trithorax group factors from *bxd***PRE****

**Fig. 2:**
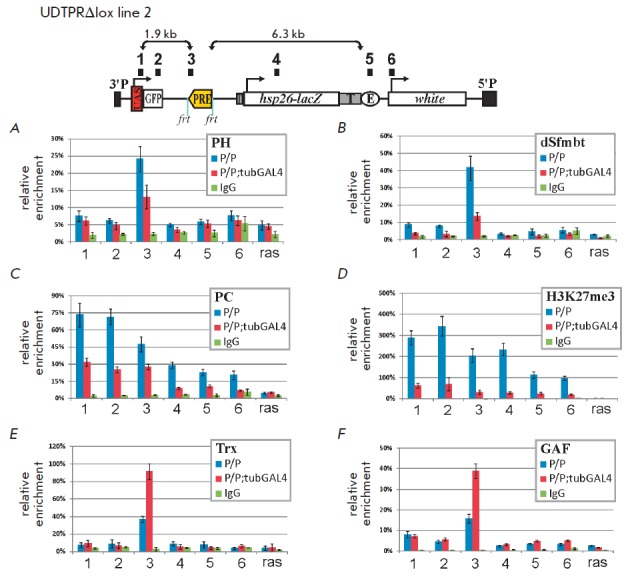
Analysis of PcG/TrxG recruitment during transcriptional read-through. X-ChIP experiment with chromatin isolated from adult flies was performed. Numbers on top of the constructs (1, 2, 3, 4, 5 and 6) indicate the primer pairs used for qPCR. X-ChIP results are presented as a percentage of Iput sample normalized to the endogenous positive control, region adjacent to 660 bp bxdPRE in the genome. The coding part of the Ras64B gene was used as a negative control (ras). Blue bars on the diagrams indicate relative X-ChIP signal levels in homozygote lines (P/P), red bars indicate relative X-ChIP signal levels in homozygote lines expressing GAL4 (P/P; tubGAL4), and green bars indicate signal levels obtained using nonspecific antibodies. Vertical lines indicate SDs. X-ChIP experiments were performed with antibodies against PH (A), dSfmbt (B), PC (C), H3K27me3 (D), Trx (E), and GAF (F)

We have previously shown that even robust transcription does not lead to complete elimination of PcG/TrxG complexes from *bxd*PRE if it is oriented forward in the transgene. We tested the influence of transcriptional read-through in the case of reverse orientation of* bxd*PRE. For this purpose, we conducted immunoprecipitation of chromatin isolated from adult homozygous flies in the presence or absence of GAL4 (*Fig. 2*). Immunoprecipitation was carried out using samples obtained from the transgenic line UDTPR (№ 2) with a deleted SV40 transcription terminator. Six areas of the construct were used for PCR analysis: 1 –UAS promoter, 2 –*eGFP *gene coding region, 3 –*bxd*PRE, 4 –*LacZ *gene coding region, 5 –*white *gene enhancer, and 6 –*white *gene promoter. As a positive control, we used the genomic region of *bxd*PRE adjacent to the element utilized in transgenic constructs, while the coding region of the *Ras64B *gene was used as a negative control (*Fig. 2*).

It has been shown that the peak of the PH (PRC1 complex, *Fig. 2A*) and dSfmbt (PhoRC complex, *Fig. 2B*) factors recruitment corresponds to *bxd*PRE in the transgene. Localization of these factors is consistent with the data according to which PH and dSfmbt are found predominantly in PRE elements but not in other regions of the repressed domain [14, 15, 21, 39, 40].

The level of recruitment of these factors decreases upon induction of transcription through *bxd*PRE, but they are not eliminated completely. A similar result was obtained when analyzing the impact of transcription on the recruitment of the PH and dSfmbt factors to *bxd*PRE located in transgene in forward orientation relative to the UAS promoter [21].

Factor PC of the PRC1 complex specifically interacts with histone 3 trimethylated at lysine 27 (H3K27me3) [41, 42], a modification characteristic of PcG-repressed chromatin [16, 40]. Recruitment of the PC factor, as well as H3K27me3, contrary to other core components of PcG complexes, is not limited to PRE and covers a wider area subjected to repression [16, 21, 40, 43]. In agreement with this, a wider profile of distribution of the PC factor (*Fig. 2C*) and H3K27me3 modification (*Fig. 2D*) has been found in the derivative of the UDTPR transgene. Introduction of a GAL4 activator did not lead to complete elimination of PC and H3K27me3, but there was a significant decrease in the level of their recruitment to *bxd*PRE and the surrounding areas of the transgene.

We also analyzed the recruitment of the TrxG factors Trx (*Fig. 2E*) and GAF (*Fig. 2F*). It was established that the induction of transcription through *bxd*PRE leads to a 2-fold increase in the recruitment of both factors to *bxd*PRE.

Thus, transcription through PRE leads to a change in the level of PcG/TrxG factors recruitment but not to complete displacement of these proteins from DNA.

## CONCLUSIONS

The repression/activation of various *Drosophila *genes requires PcG/TrxG proteins [1-4] that bind to the DNA elements termed PREs [5, 6]. A series of studies has shown that a lack of PRE-mediated repression correlates with the presence of non-coding transcripts [24, 44]. On this basis, a model was proposed according to which transcriptional read-through physically dislodges PRE-associated factors and replaces repressive histone modifications with active ones [24]. Despite its apparent clarity, this hypothesis has not been tested directly.

On the other hand, according to other data, non-coding RNAs from *Ubx *locus (lncRNA-*bxd *and lncRNA iab-8) are associated with the domain subjected to repression [45, 46]. Moreover, in spite of scrupulous studies, non-coding RNAs have not been detected in the regions of PRE elements of several loci (*invected, engrailed*), which indicates the absence of a key role for transcription, at least in the functioning of several PRE elements [47].

Previously, we tested the effect of transcription on GAL4-mediated activity switch of PRE [21]. As a result, we found that even robust transcription through* bxd*PRE does not lead to complete elimination of PcG/ TrxG factors but changes the ratio in the binding of these proteins: recruitment of PcG decreases, while the recruitment of TrxG increases. The transcriptional effect was analyzed in detail for *bxd*PRE incorporated into transgene in direct orientation [21]. At the same time, active and inactive states of PRE in *vg *locus correlate with transcriptional read-through from different DNA strands [48]. Therefore, the direction of transcription through PRE can potentially be crucial for the activity of PRE. We have tested this possibility and found that alteration of *bxd*PRE orientation does not lead to a change in the transcriptional read-through effect. Recruitment of PcG/TrxG factors is not abolished upon transcription. However, the recruitment of the TrxG proteins Trx and GAF increases, while the recruitment of PcG proteins (PH, dSfmbt, PC) decreases.

The presence of the strong terminator SV40 between the UAS promoter and *bxd*PRE also does not prevent abolition of repression. Apparently, the GAL4-binding sites themselves are capable of neutralizing PRE-mediated repression and transcription though PRE does not play a crucial role in this process.

PRE elements regulate genes the expression of which is changed during differentiation and development. Thus, a particular gene must be expressed in certain cells at a certain stage of development, and then its expression should be suppressed. Apparently, the recruitment of repressor factors to PRE in activating state could be required for the quick PRE activity switch to the repressing state and to abort the expression of the target gene at a certain moment in time. A logically similar mechanism has been described for many eukaryotic promoters: pausing of RNA polymerase II. In this case, RNA polymerase II binds to a transcriptionally inactive promoter and, if necessary, quickly triggers transcription.

The mechanism that allows the recruitment of proteins to PRE during transcriptional read-through is unclear. A series of DNA-binding factors with zinc finger motifs are known to be associated with PREs. It is possible that transcription does not interfere with direct DNA-protein contacts. On the other hand, there is a possibility that retention of complexes at PRE during transcriptional read-through is mediated by the contacts between the PcG/TrxG factors and histone proteins. In accordance, PcG proteins contain domains capable of interacting directly with nucleosomes (for example, the MBT domains of dSfmbt and Scm) [14, 49, 50] and transcription does not result in complete dissociation of nucleosomes [51]. However, the details of these processes are currently unclear and require further investigation.

## Abbreviations

PcG: Polycomb group proteins
TrxG: Trithorax group proteins
bxd: bithoraxoid
